# microRNA-20a Inhibits Autophagic Process by Targeting ATG7 and ATG16L1 and Favors Mycobacterial Survival in Macrophage Cells

**DOI:** 10.3389/fcimb.2016.00134

**Published:** 2016-10-18

**Authors:** Le Guo, Jin Zhao, Yuliang Qu, Runting Yin, Qian Gao, Shuqin Ding, Ying Zhang, Jun Wei, Guangxian Xu

**Affiliations:** ^1^Ningxia Key Laboratory of Clinical and Pathogenic Microbiology, General Hospital of Ningxia Medical UniversityYinchuan, China; ^2^Department of Medical Laboratory, School of Clinical Medicine, Ningxia Medical UniversityYinchuan, China; ^3^Ningxia Key Laboratory of Clinical and Pathogenic MicrobiologyYinchuan, China; ^4^Clinical Laboratory, General Hospital of Tianjin Medical UniversityTianjin, China; ^5^Medical School of Nantong University, Nantong UniversityNantong, China; ^6^Department of Molecular Microbiology and Immunology, Bloomberg School of Public Health, Johns Hopkins UniversityBaltimore, MD, USA

**Keywords:** microRNA-20a, ATG7, ATG16L1, autophagy, *M. tuberculosis*

## Abstract

Autophagy plays important roles in the host immune response against mycobacterial infection. *Mycobacterium tuberculosis* (*M. tuberculosis*) can live in macrophages owing to its ability to evade attacks by regulating autophagic response. MicroRNAs (miRNAs) are small noncoding, endogenously encoded RNA which plays critical roles in precise regulation of macrophage functions. Whether miRNAs specifically influence the activation of macrophage autophagy during *M. tuberculosis* infection are largely unknown. In this study, we demonstrate that BCG infection of macrophages resulted in enhanced expression of miRNA-20a, which inhibits autophagic process by targeting ATG7 and ATG16L1 and promotes BCG survival in macrophages. Forced overexpression of miR-20a decreased the expression levels of LC3-II and the number of LC3 puncta in macrophages, and promoted BCG survival in macrophages, while transfection with miR-20a inhibitor had the opposite effect. Moreover, the inhibitory effect of miR-20a on autophagy was further confirmed by transmission electron microscopy (TEM) analysis. Quantification of autophagosomes per cellular cross-section revealed a significant reduction upon transfection with miR-20a mimic, but transfection with miR-20a inhibitor increased the number of autophagosomes per cellular cross-section. Moreover, silencing of ATG7 significantly inhibited autophagic response, and transfection with ATG7 siRNA plus miR-20a mimic could further decrease autophagic response. Collectively, our data reveal that miR-20a inhibits autophagic response and promotes BCG survival in macrophages by targeting ATG7 and ATG16L1, which may have implications for a better understanding of pathogenesis of *M. tuberculosis* infection.

## Introduction

Autophagy is an evolutionarily conserved mechanism responsible for intracellular degradation of proteins and whole organelles and for resistance to pathogenic infection (Yang and Klionsky, 2010; Deretic et al., 2013). The autophagic process involves the formation of autophagosomes which are double membrane vesicles. Autophagosomes engulf portions of cytoplasm together with proteins, whole organelles or pathogens such as *Mycobacterium tuberculosis* (*M. tuberculosis*), become mature gradually along the endocytic pathway, acidify, and finally form autolysosomes by fusing with lysosome, and bring about degradation of inclusions in autophagic vacuoles. *M. tuberculosis* is a highly successful human pathogen, which represents the leading bacterial cause of death worldwide (Korbel et al., 2008; Russell et al., 2010). *M. tuberculosis* can reside in macrophages, and avoid elimination by inhibiting the acidification of phagocytotic vesicles or by other means (Gengenbacher and Kaufmann, 2012). Mycobacterial lipids have been found to induce autophagy and activate mTOR signaling, and BCG has an innate ability to reduce the macrophage autophagy response (Zullo and Lee, 2012). *M. tuberculosis* “enhanced intracellular survival” (eis) gene can inhibit host innate immune defenses by modulating autophagy, inflammation, and cell death through redox-dependent signaling (Shin et al., 2010). The autophagic process is closely related to numerous autophagy-related proteins (ATGs) (Weidberg et al., 2011). Among the ATGs, ATG7 and ATG16L1 are essential for autophagy. ATG7 participates in two important functions involved in autophagosome formation and in vesicle progression. ATG7 gene knockout mice die within 1 day from birth on account of an impaired autophagy pathway (Komatsu et al., 2005). ATG16L1 interacts with ATG12-ATG5 to mediate the conjugation of phosphatidylethanolamine (PE) to LC3, to produce a membrane-bound activated form of LC3 named LC3-II. Therefore, ATG16L1 controls the elongation of the nascent autophagosomal membrane (Levine et al., 2011).

microRNAs (miRNAs) are small noncoding, endogenously encoded RNAs which are about 22 nucleotides in length. More than 60% of all mammal protein-coding genes are regulated by miRNAs (Chekulaeva and Filipowicz, 2009). miRNAs can depress protein synthesis by binding mRNA in their 3′-UTR or bringing about mRNA degradation (Yates et al., 2013). MiRNAs are involved in a wide variety of biological processes such as immune regulation (Sayed and Abdellatif, 2011). Recently, an increasing number of miRNAs have been demonstrated to play a certain role in autophagy by regulating ATGs or their regulators, especially in cancer (Zheng et al., 2015; Rothschild et al., 2016). However, the regulatory mechanisms of miRNAs on autophagy during *M. tuberculosis* infection is largely unknown. miR-20a is a member of the miR-17-92 cluster which encodes for six individual miRNAs including miR-17, miR-18a, miR-19a, miR-20a, miR-19b-1, and miR-92a. Studies have shown that miR-20a inhibits autophagy in both hypoxia-induced osteoclast differentiation (Sun et al., 2015) and ischemic kidney injury (Wang I. K. et al., 2015) by targeting ATG16L1. Furthermore, miR-20a has been shown to negatively regulate autophagy by targeting RB1CC1/FIP200 in breast cancer cells (Li S. et al., 2016) and inhibit autophagy induced by leucine deprivation via suppression of ULK1 expression in C2C12 myoblasts (Wu et al., 2012).

In this study, we investigated the potential role of miR-20a in regulating autophagy and bacterial clearance in macrophages. We demonstrated that miR-20a is significantly induced in RAW264.7 cells infected with BCG or treated with rapamycin and that overexpression of miR-20a inhibited antophagy process, thus depressing antimicrobial response during mybobacterial infection by targeting ATG7 and ATG16L1. These findings provide a better understanding of pathogenesis of *M. tuberculosis* infection.

## Materials and methods

### Mycobacterial culture

Bacillus Calmette-Guérin (BCG) Beijing strain was purchased from the Center for Disease Control and Prevention (CDC) of China. The BCG bacilli were grown in Middlebrook 7H9 (Goybio, China) broth containing 10% albumin dextrose catalase (ADC) supplement at 37°C for 2 weeks. Then the BCG bacilli were harvested by centrifugation at 500 × g for 10 min, and resuspended in culture medium.

### High-throughput sequencing of small RNAs

Murine macrophage RAW264.7 cells were treated with 3-methyladenine (3-MA, 5 mM) or rapamycin (Rapa, 50 μg/ml). Total RNA was isolated by using TRIzol reagent (Sigma). The small RNA libraries were constructed by using TruSeq Small RNA Sample Preparation Kit (Illumina). Small RNAs were ligated first with the 5′ RNA adaptor and then with the 3′ RNA adaptor. After first-strand synthesis and PCR amplification, the final bands were purified and submitted for sequencing on Illumina HiSeq2500 analyzer. Sequencing was performed at Biomarker Technologies (Beijing). After sequsencing, the reads went through the data cleaning procedure including filtering the low quality reads, removing reads containing unknown bases greater than 10%, filtering primer adaptor sequences, triming adaptor contaminations, and retaining only trimmed reads of sizes from 18 to 30 nt.

### Bioinformatics analysis of small RNA sequences

The microRNA expression profiles in RAW264.7 treated with 3-methyladenine (3-MA) or rapamycin were detected by high-throughput sequencing. The raw sequencing data were mapped to the mouse genome (ftp://ftp.ensembl.org/pub/release-78/fasta/mus_musculus/) by using miRDeep2 (https://www.mdc-berlin.de/8551903/en/research/research_teams/systems_biology_of_gene_regulatory_elements/projects/miRDeep). Reads perfectly matching the mouse genome were used for further analysis. miRNA expression data were mean centered and represented by a heat map by using Multi Experiment Viewer software (MeV, http://mev–multiple-experiment-viewer.sharewarejunction.com/). To visualize interaction network of miRNA regulating ATGs, ATGs were selected in terms of GO biological processes. The list of ATGs was further filtered by using the potential target protein information of 19 selected miRNAs. Eventually, 20 ATGs were selected. For miRNAs, 7 miRNAs (miR-20a, miR-92a, miR-152, miR-210, miR-449a, miR-96, miR-182) with fold changes (3-MA/NC or Rapa/NC) greater than 1.5 or lower that 0.5 were selected, and 4 other miRNAs (miR-17, miR-18a, miR-19a, miR-19b) in miR-17-92 cluster were also selected. Additionally 8 miRNAs (miR-10b, miR-30b, miR-144, miR-155, miR-21, miR-29a, miR-181a, miR-125a) known to be important in autophagy were selected. The miRNA-autophagy-related protein interaction network was drawn by cytoscape 3.4. The potential target genes of miR-20a were predicted by miRWalk (http://zmf.umm.uni-heidelberg.de/apps/zmf/mirwalk/micrornapredictedtarget.html), miRDB (http://www.mirdb.org/miRDB/), miRanda (http://www.microRNA.org/) and Targetscan (http://www.targetscan.org/) softwares respectively, and the venn diagram was drawn by Venny 2.1 software (http://bioinfogp.cnb.csic.es/tools/venny/).

### Transient transfection

Murine macrophage RAW264.7 cells were transiently transfected with 50 nM miR-20a control or miR-20a mimic (GenePharma, Shanghai, China); 50 nM control or miR-20a inhibitor (GenePharma, Shanghai, China); or 50 pmol ATG16L1 siRNA; or 50 pmol ATG7 siRNA; or 1.5 μg plasmid, using Lipofectamine 2000 (Invitrogen, USA) according to the manufacturer's instructions.

### Luciferase reporter assays

For luciferase reporter assays, the wild-type 3′UTRs of ATG16L1 and ATG7, which contain the binding elements of miR-20a, were obtained by PCR. Then the mutant 3′UTRs of ATG16L1 and ATG7 were obtained by site-directed mutagenesis. The primers used during the study were as follows: wild-type ATG16L1 3′UTR (Forward, 5′-CGACGCGCAGAACCT GAACTCCCTT-3′; Reverse, 5′-TCAACTTCCAACGCCA CAACCCAAGCTTGGG-3′), mutant ATG16L1 3′UTR (Forward, 5′-CGACGCGCAGAACCTGAACTCCCTT-3′; Reverse, 5′-TAACGCTCAAAGTAAGTGTGACCCAAGCTTGGG-3′), wild-type ATG7 3′UTR (Forward, CGACGCGTACCATCTGTGCAAGGCTCC; Reverse, 5′-ACTCCATGACAACACTGCGGTCCCAAG CTTGGG-3′), and mutant ATG7 3′UTR (Forward, 5′-CGACGCGTACCATCTGTGCAAGGCTCC-3′; Reverse, 5′-AATGCGTTCTTAAACCGAGGCTGCCCAAGCTT GGG-3′). These above-mentioned wild-type or mutated 3′UTR fragments were then cloned into pMIR-Report vector (Ambion), producing pMIR-Report-WT-ATG16L1 (harboring wild-type 3′UTR of ATG16L1; ATG16L1-WT), pMIR-Report-Mut-ATG16L1 (harboring mutant 3′UTR of ATG16L1; ATG16L1-Mut), pMIR-Report-WT-ATG7 (harboring wild-type 3′UTR of ATG7; ATG7-WT) and pMIR-Report-Mut-ATG7 (harboring mutant 3′UTR of ATG7; ATG7-Mut). Plasmids ATG16L1-WT, ATG16L1-Mut, ATG7-WT or ATG7-Mut were cotransfected with miR-20a control or miR-20a mimic into 293T cells with Lipofectamine 2000 (Invitrogen). The relative activity of firefly luciferase unit (RLU) at 48 h post-transfection was determined by the Dual-Luciferase Reporter Assay System (Promega) following the manufacturer's protocol.

### Quantitative real-time PCR

The RAW264.7 cells were treated with rapamycin, 3-MA or BCG, and the expression levels of miRNAs in miR-17-92 cluster were measured by using qRT-PCR analysis. Total RNA was isolated by using TRIzol reagent (Sigma). miRNAs were purified by RNAiso for Small RNA kit (Takara). For miRNAs, cDNA was synthesized by using TransScript First-Strand cDNA Synthesis SuperMix Kit (TransGen Biotech). Quantitative real-time PCR (qRT-PCR) was performed by using TransStart Top Green qPCR SuperMix kit (TransGen Biotech). Small nuclear RNA (RNU6) was used for normalization. The primers for qRT-PCR are shown in Supplementary Table 1.

A qRT-PCR assay was used to quantify the BCG load in RAW264.7 cells following a treatment with miR-20a control, miR-20a inhibitor, miR-20a mimic or ATG7 siRNA. Briefly, the RAW264.7 cells were transfected with miR-20a control, miR-20a mimic, miR-20a inhibitor, ATG7 siRNA for 24 h, and then infected with BCG for 24 h. The bacterial load was determined by assessing IS6110 DNA sequence specific for BCG by qPCR assay. The primers for IS6110 were 5′-GGACGGAAACTTGAACACG-3′ (forward) and 5′-TCTGACGACCTGATGATTGG-3′ (reverse). Standard PCR cycle parameters were as follows: 95°C for 30 s, followed by 40 cycles of 95°C for 15 s, 60°C for 30 s and 72°C for 30 s. The quantity of BCG IS6110 is normalized for the DNA content of RAW264.7 cells with primers targeting the murine β-actin, using a comparative Ct (ΔΔCt) method.

### Western-blot analysis

For Western blot analysis, proteins were loaded onto 12 or 15% SDS-PAGE gels and transferred to a polyvinylidene difluoride membrane (PVDF, Millipore, USA). Membranes were incubated with anti-ATG16, anti- ATG7, anti-LC3, and anti–β-actin. Immunoreactive band analysis was performed by using ECL reagent (Thermo Fisher). Densitometric analysis of bands was conducted by using ImageJ software.

### Confocal microscopy

The inhibitory effects of miR-20a on autophagy were evaluated by counting LC3 puncta in RAW264.7 cells after BCG infection. The RAW264.7 cells were transfected with miR-20a control, miR-20a mimic, miR-20a inhibitor, ATG7 siRNA, ATG7 siRNA plus rapamycin, ATG7 siRNA plus miR-20a mimic, and then treated with BCG at an MOI of 10 for 24 h. The RAW264.7 cells were fixed with 4% paraformaldehyde. The RAW264.7 cells were blocked with 3% bovine serum albumin (BSA) and incubated with 10 μg/ml Rabbit anti-LC3 primary antibody (Cell Signaling Technology, Inc.) and then 2 μg/ml Alexa Fluor 488–conjugated goat anti-rabbit IgG (Abcam) before mounting. The images of cells were visualized and acquired using an Olympus DSU spinning disk confocal microscope under a 100 × objective oil lens. The number of endogenous LC3 punctate dots was counted by using ImageJ Software version 1.46. At least 15 cells per experimental group were counted and each condition was assayed in triplicate. The LC3-II protein levels were evaluated by Western blot using Rabbit anti-LC3 primary antibody (Cell Signaling Technology, Inc.) and HRP-conjugated Goat anti-Rabbit IgG secondary antibody (Proteintech Group, Inc.).

### Transmission electron microscopy

Transmission electron microscopy (TEM) was used to detect autophagosomes as previously described (Hayashi-Nishino et al., 2009; Frankel et al., 2011). The RAW264.7 cells were transfected with miR-20a control, miR-20a mimic, miR-20a inhibitor or miR-20a mimic plus ATG7 siRNA for 24 h, and then infected with BCG for 24 h. The RAW264.7 cells were fixed in 2% v/v glutaraldehyde in 0.05 M sodium phosphate buffer for 24 h. After washing 3 times, the samples were post-fixed in 1% w/v OsO4 in 0.12 M sodium cacodylate buffer for 2 h. The ethanol solutions with different concentrations were used to dehydrate the samples. After that, the samples were transferred to propylene oxide and embedded in Epon. The uranyl acetate and lead citrate were used to stain the sections with about 80 nm thick. Imaging was performed by a Phillips CM 100 BioTWIN transmission electron microscope (× 3000 for close-ups). ITEM digital imaging software was utilized to obtain highresolution images of cellular cross-sections which are suitable for identifying and counting autophagosomes. For each experimental group, 20 cellular cross-sections were examined.

### Statistical analysis

All of the data are presented as mean ± SD of independent experiments. Statistical analyses were performed using two-tailed Student's *t*-test. Comparisons between groups were performed using ANOVA. Significant differences were assigned to *p* < 0.05, <0.01, and <0.001, denoted by ^*^, ^**^, and ^***^, respectively.

## Results

### High-throughput sequencing of small RNA

The results of sequencing analysis showed that 90 miRNAs were increased or decreased significantly in RAW264.7 treated with 3-MA or rapamycin compared with untreated RAW264.7 cells (Supplementary Data Sheet 1). The heat map of the alteration of miRNAs is shown in Supplementary Figure 1A. Among the 90 miRNAs, 52 miRNAs were increased more than 1.5-fold or decreased more than 2-fold in RAW264.7 cells treated with 3-MA or rapamycin compared with normal RAW264.7 cells (Supplementary Figure 1B). Several miRNAs in miR-17-92 cluster have been found to take part in autophagy regulation in other cell models, such as miR-17 (Kumar et al., 2016), miR-18a (Fan et al., 2016), miR-19a (Gao et al., 2016), miR-20a (Li S. et al., 2016), indicating that miRNAs in miR-17-92 cluster may also regulate autophagy in macrophages. The results of high-throughput sequencing demonstrated that the expression level of miR-20a in miR-17-92 cluster was increased in RAW264.7 treated with rapamycin but was reduced in RAW264.7 treated with 3-MA (Supplementary Figure 1C). The target genes of miRNA were predicted by miRWalk, miRDB, miRanda, and Targetscan. After analysis by Venny 2.1 software, 432 potential target genes of miR-20a were obtained. Among the 432 potential target genes, only two target genes (ATG7 and ATG16L1) were related to autophagy. In order to define the relationship between 19 selected miRNAs and 20 filtered ATGs, a circulatory biological network was drawed by cytoscape software (Supplementary Figure 1D). Among the 19 selected miRNAs, 9 miRNAs (miR-20a, miR-152, miR-210, miR-449a, miR-96, miR-182, miR-181a, miR-155, and miR-125a) with significant change had been demonstrated to have a specific target protein in those 20 filtered ATGs. 3 miRNAs (miR-30b, miR-144, and miR-17) known to have specific target proteins in those 20 filtered ATGs have no significant change or weren't detected by high-throughput sequencing. 3 miRNAs (miR-92a, miR-21, and miR-19b) with significant change after detection by high-throughput sequencing analysis had several potential target proteins in those 20 filtered ATGs. 4 miRNAs (miR-10b, miR-19a, miR-18a, and miR-29a), which were no significant change or weren't detected by high-throughput sequencing, had also specific target proteins in those 20 filtered ATGs.

### miR-20a expression in RAW264.7 after BCG infection

**Figure 1:**
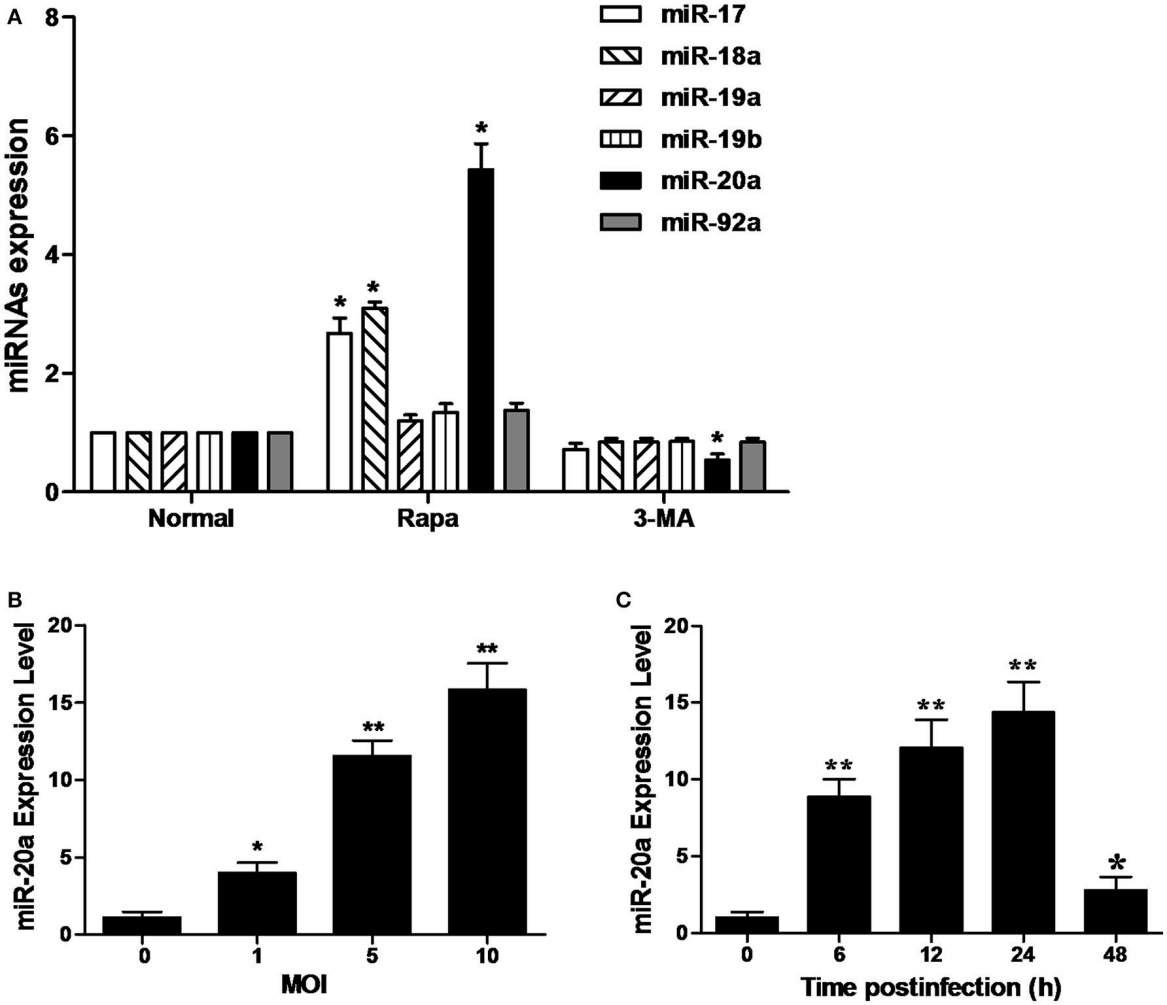
** MiR-20a expression is induced after mycobacterial infection or treatment with rapamycin (Rapa). (A)** The expression levels of miRNAs in miR-17-92 cluster. The RAW264.7 cells were treated with 50 μg/ml rapamycin for 2 h or 10 mM 3-MA for 12 h. The expression levels of miR-17, miR18a, miR-19a, miR-19b, miR-20a, and miR-92a were determined by qRT-PCR. **(B)** RAW264.7 cells were infected with BCG at different MOIs for 24 h. The expression levels of miR-20a were determined by qRT-PCR. **(C)** RAW264.7 cells were infected with BCG at an MOI of 10 for the indicated time points. The expression levels of miR-20a were determined by qRT-PCR. Data represent the means ± SD from three independent experiments. ^*^p < 0.05, ^**^p < 0.01.

As shown in Figure 1A, the RAW264.7 cells treated with rapamycin showed a significant increase in the expression of miR-20a, miR-17, and miR-18a. However, only miR-20a was reduced in the RAW264.7 cells treated with 3-MA. This result implied that miR-20a might be involved in the autophagy process. Then, we determined miR-20a expression significantly increased in RAW264.7 cells after BCG infection. BCG-infected RAW264.7 cells displayed a gradual increase in expression of miR- 20a in a dose- and time-dependent manner (Figures 1B,C).

### miR-20a suppresses ATG7 and ATG16L1 by interacting with their 3′UTR

**Figure 2:**
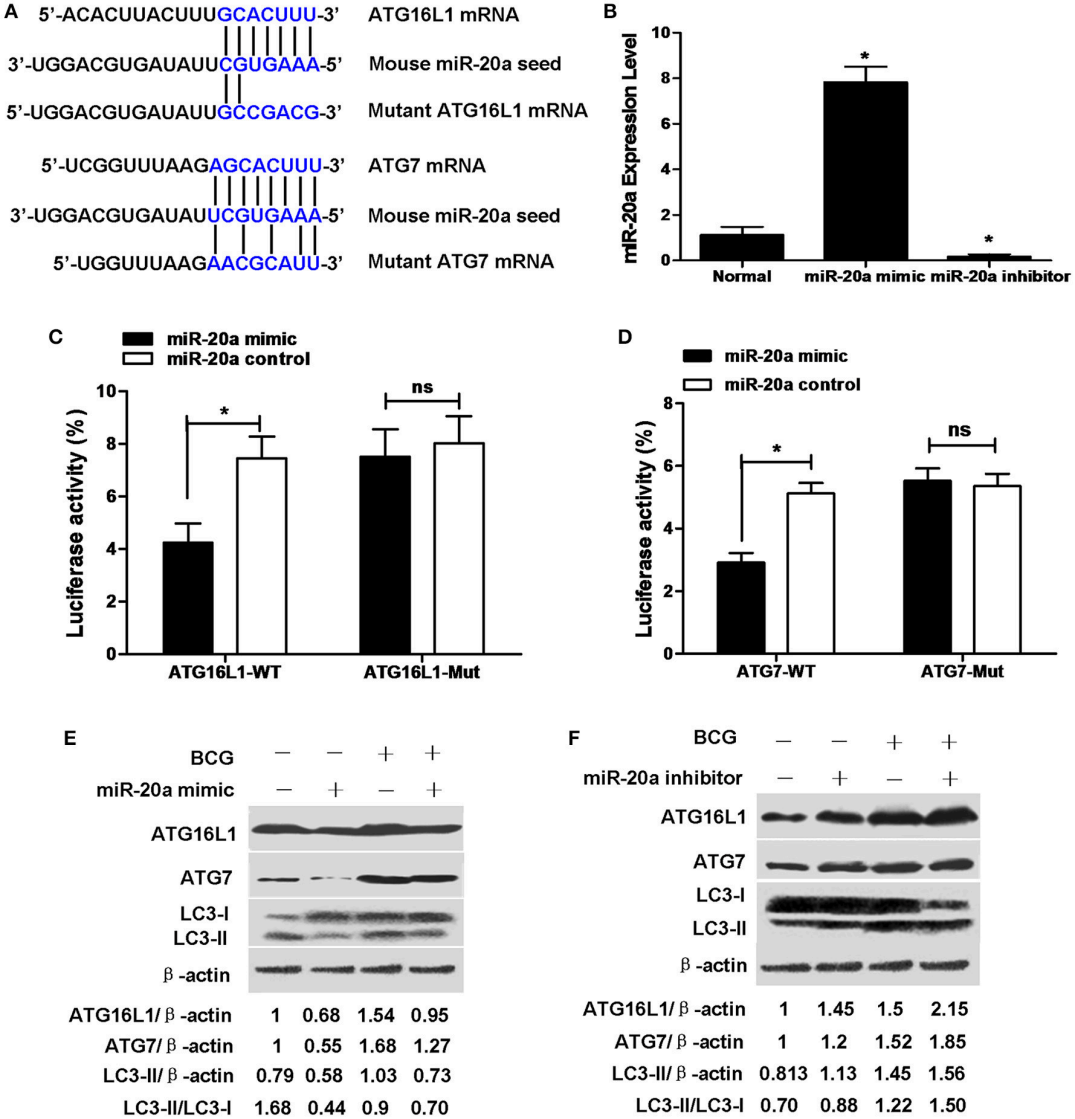
**miR-20a can reduce the protein expression of ATG7 and ATG16L1 in RAW264.7 cells by targeting their 3′UTRs. (A)** The seed sequences of human and mouse miR-20a were predicted by bioinformatic analysis. The sequence of the 3′UTR seed mutant of ATG7 and ATG16L1 used for the reporter assays and the predicted disruption of the miR-20a interaction was also shown. **(B)** The 293T cells were transfected with miR-20a mimic or miR-20a inhibitor. The expression levels of miR-20a were measured by qRT-PCR. Data represent the means ± SD from four independent experiments. ^*^p < 0.05. **(C)** The 293T cells were transfected with miR-20a control, miR-20a mimic and a wild-type (ATG16L1-WT) or mutant ATG16L1 3′UTR (ATG16L1-Mut) luciferase reporter plasmid, and the luciferase activity of the 293T cells was assessed at 24 h after transfection. Data represent the means ± SD from four independent experiments. ^*^p < 0.05. **(D)** The 293T cells were transfected with miR-20a control, miR-20a mimic and a wild-type (ATG7-WT) or mutant ATG7 3′UTR (ATG7-Mut) luciferase reporter plasmid, and the luciferase activity of the 293T cells was assessed at 24 h after transfection. Data represent the means ± SD from four independent experiments. ^*^p < 0.05. **(E,F)** RAW264.7 cells were transfected with miR-20a mimic or inhibitor and then infected with BCG at an MOI of 10 for 24 h. The expression levels of ATG16L1, ATG7, LC3-I, and LC3-II were detected by Western-blot. The values of ATG16L1/β-actin, ATG7/β-actin, LC3-II/β-actin, and LC3-II/LC3-I were labeled below the representative blot.

ATG16L1 and ATG7 showed potential target sequences of miR-20a by using miRanda algorithm and TargetScan analysis, which matched with miR-20a in their 3′UTR (Figure 2A). We demonstrated that overexpression of miR-20a inhibited luciferase activity in the 293T cells containing the ATG16L1-WT or ATG7-WT reporter, but failed to inhibit luciferase activity in the 293T cells containing the ATG16L1-WT or ATG7-Mut reporter (Figures 2C,D). In addition, qRT-PCR data demonstrated that transfection with miR-20a mimic significantly increased the expression levels of miR-20a. However, transfection with miR-20a inhibitor significantly reduced the expression levels of miR-20a in 293T cells (Figure 2B).

To further study whether miR-20a inhibits endogenous ATG16L1 and ATG7, RAW264.7 cells were transfected with control, miR-20a mimic or miR-20a inhibitor, the expression levels of ATG16L1 and ATG7 were detected by Western blot. The results demonstrated that transfection with an miR-20a mimic resulted in a significant decrease in ATG16L1 and ATG7 protein expression in uninfected and BCG-infected RAW264.7 cells (Figure 2E). However, transfection with an miR-20a inhibitor brought about a significant increase in uninfected and BCG-infected RAW264.7 cells (Figure 2F). Taken together, these results indicate that miR-20a could inhibit the expression of ATG16L1 and ATG7 by directly interacting with their 3′UTR binding sites. Moreover, transfection with miR-20a mimic significantly decreased the levels of LC3-II/LC3-I or LC3-II/β-actin in RAW264.7 cells before and after BCG infection (Figure 2E), but transfection with miR-20a inhibitor increased the levels of LC3-II/LC3-I or LC3-II/β-actin in the uninfected and BCG-infected RAW264.7 cells (Figure 2F).

### miR-20a inhibits autophagy in macrophages

**Figure 3:**
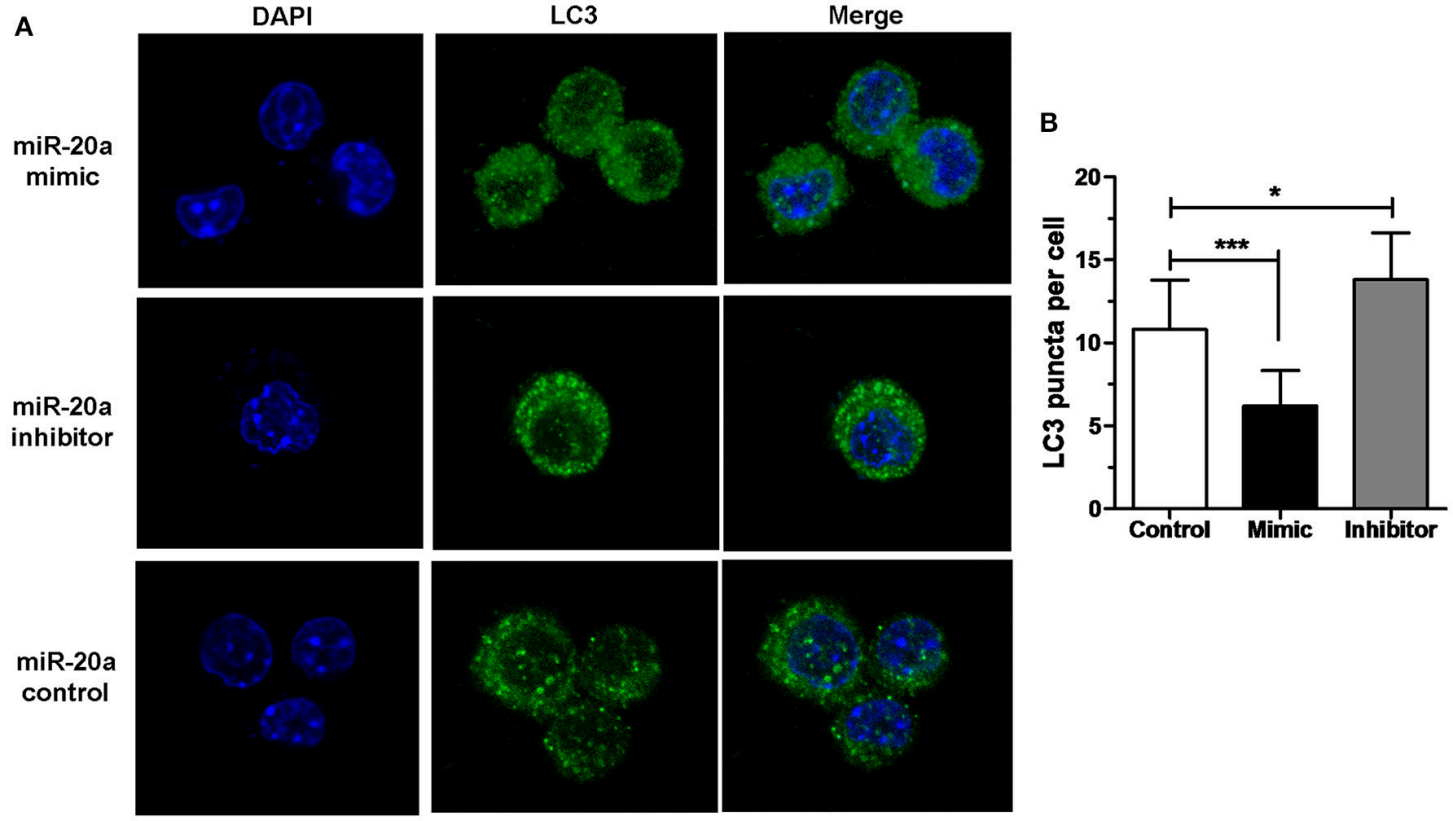
**miR-20a inhibited autophagosome formation. (A)** The RAW264.7 cells were transfected with miR-20a control, miR-20a mimic, or miR-20a inhibitor for 24 h, and then treated with BCG at an MOI of 10 for 24 h. After that, the RAW264.7 cells were fixed and incubated with rabbit anti-LC3 antibody, followed by Alexa Fluor 488–conjugated goat anti-rabbit IgG. LC3 puncta formation was then detected by confocal microscopy. **(B)** The data were quantified by counting the number of autophagosomes per cross-sectioned cell (Control, *n* = 15; Mimic, *n* = 15; Inhibitor, *n* = 20). Data represent the means ± SD from three independent experiments. ^*^p < 0.05, ^***^p < 0.001.

The results of immunofluorescence analysis showed that transfection with miR-20a mimic significantly decreased the number of LC3 puncta in RAW264.7 cells at 24 h after BCG infection compared with transfection with miR-20a control. However, transfection with miR-20a inhibitor significantly increased the number of LC3 puncta in RAW264.7 cells at 24 h after BCG infection compared with transfection with miR-20a control (Figures 3A,B). The results immunofluorescence analysis are consistent with the results of western blot about levels of LC3-II/LC3-I (Figures 2E,F).

**Figure 4:**
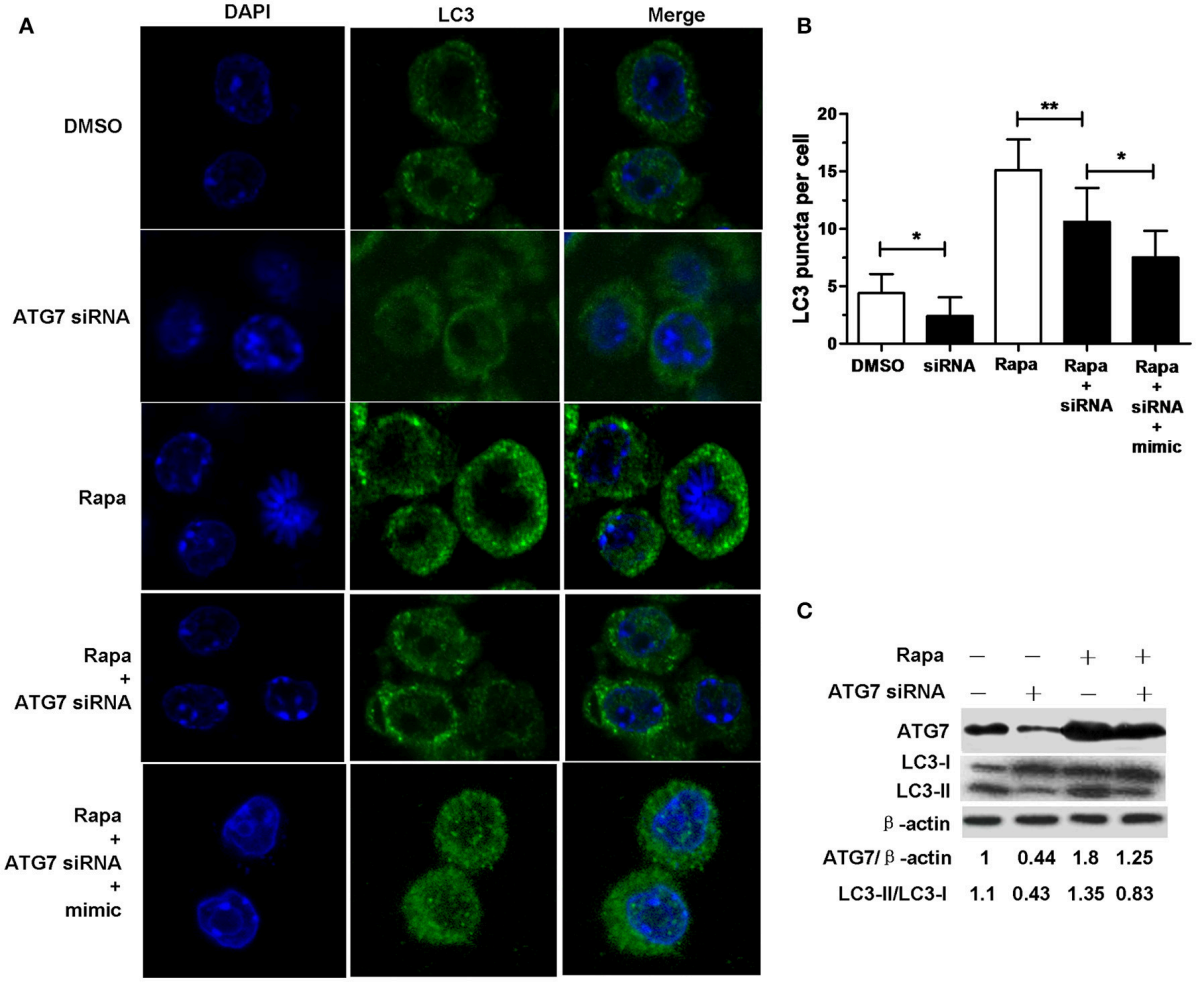
**ATG7 siRNA inhibited autophagosome formation. (A)** The RAW264.7 cells were treated with 50 μg/ml rapamycin for 2 h, and then transfected with ATG7 siRNA with or without mimic for 24 h. After that, the RAW264.7 cells were fixed and incubated with Rabbit Anti-LC3 antibody, followed by Alexa Fluor 488–conjugated goat anti-rabbit IgG. LC3 puncta formation was then detected by confocal microscopy. **(B)** The data were quantified by counting the number of autophagosomes per cross-sectioned cell (DMSO, *n* = 10; siRNA, *n* = 20; Rapa, *n* = 15; Rapa + siRNA, *n* = 20). Data represent the means ± SD from three independent experiments. ^*^p < 0.05, ^**^p < 0.01. **(C)** The RAW264.7 cells were treated with 50 μg/ml rapamycin for 2 h, and then transfected with ATG7 siRNA for 24 h. The ratio of ATG7/β-actin and LC3-II/LC3-I was determined by Western blot.

Transfection with ATG7 siRNA significantly reduced the number of LC3 puncta (Figures 4A,B), the protein expression levels of ATG7 (Figure 4C) and the amount of LC3-II/LC3-I (Figure 4C) in RAW264.7 cells with or without rapamycin, indicating that ATG7 siRNA can inhibit autophagy. More importantly, transfection with ATG7 siRNA plus miR-20a mimic further decreased the number of LC3 puncta compared to transfection with ATG7 siRNA in rapamycin-treated RAW264.7 cells (Figures 4A,B), indicating that ATG7 is only one of miR-20a targets. Collectively, these results indicates that miR-20a inhibit autophagy in macrophages.

### TEM confirms repression of autophagy by miR-20a

**Figure 5:**
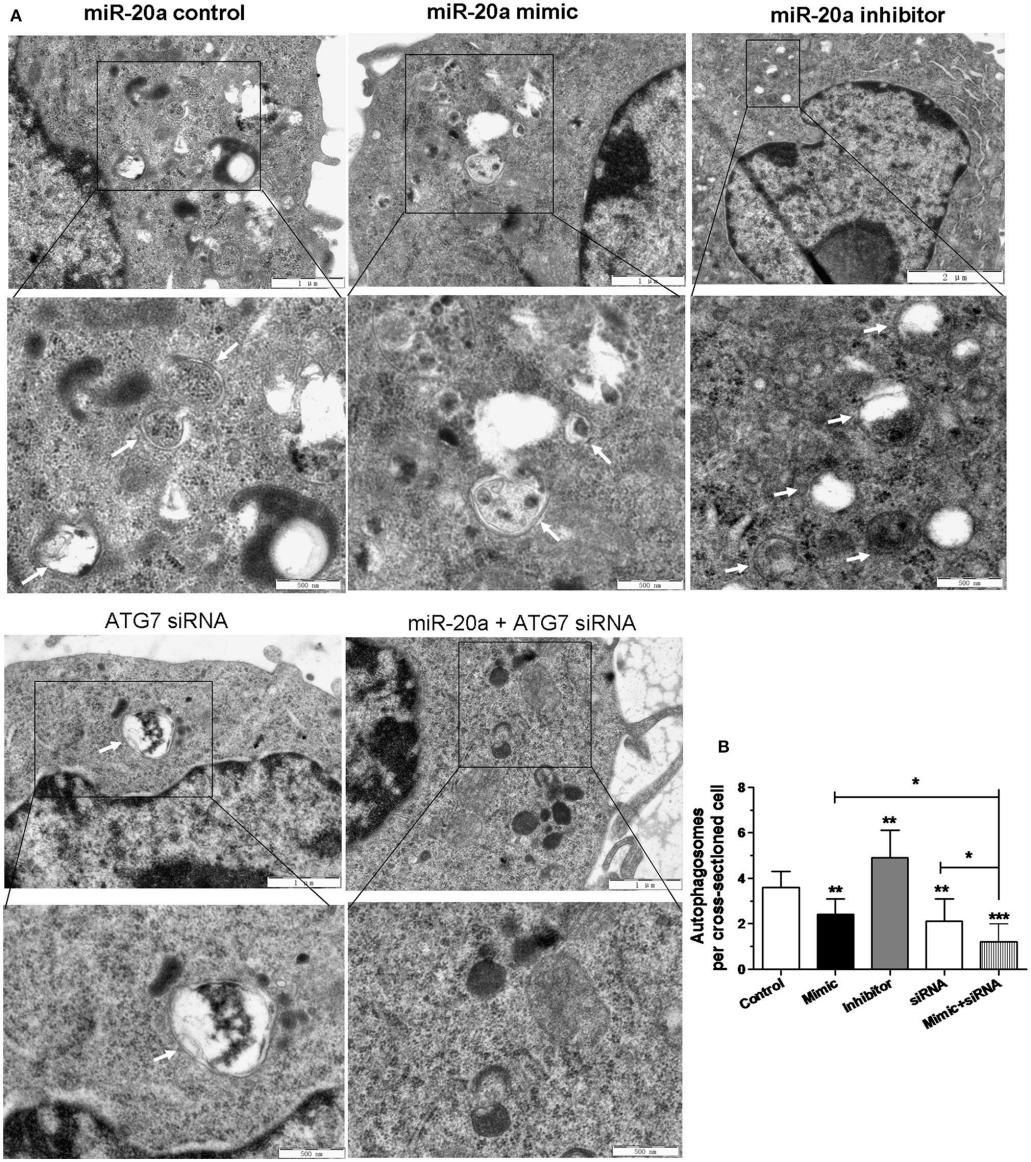
**The inhibitory effect on autophagy by miR-20a was confirmed by Transmission Electron Microscopy (TEM) detection**. The RAW264.7 cells were transfected with miR-20a control, miR-20a mimic, miR-20a inhibitor, ATG7 siRNA or ATG7 siRNA plus miR-20a for 24 h, and then infected with BCG at an MOI of 10 for 24 h. **(A)** Close-up images (× 5000 magnification) of cytoplasmic regions containing autophagosomes (denoted by black arrowheads). Scale bars represent 1 μm. **(B)** The number of autophagosomes per cross-sectioned cell was counted (20 cells per group counted by TEM). Data represent the means ± SD from three independent experiments. ^*^p < 0.05, ^**^p < 0.01, ^***^p < 0.001.

In order to confirm the inhibitory effect of miR-20a on autophagy, the autophagosomes in cellular cross-sections were detected and quantified by TEM. The representative TEM images are shown in Figure 5A. Quantification of autophagosomes per cellular cross-section revealed a significant reduction upon transfection with miR-20a mimic, ATG7 siRNA or miR-20a mimic plus ATG7 siRNA (Figure 5B), but transfection with miR-20a inhibitor increased the number of autophagosomes per cellular cross-section, confirming our results of immunofluorescence analysis (Figures 3A,B, 4A,B). Moreover, transfection with ATG7 siRNA plus miR-20a mimic further decreased the number of autophagosomes per cellular cross-section compared to transfection with ATG7 siRNA or miR-20a mimic in BCG-infected RAW264.7 cells.

### miR-20a promotes BCG survival in macrophages by inhibiting autophagy

**Figure 6:**
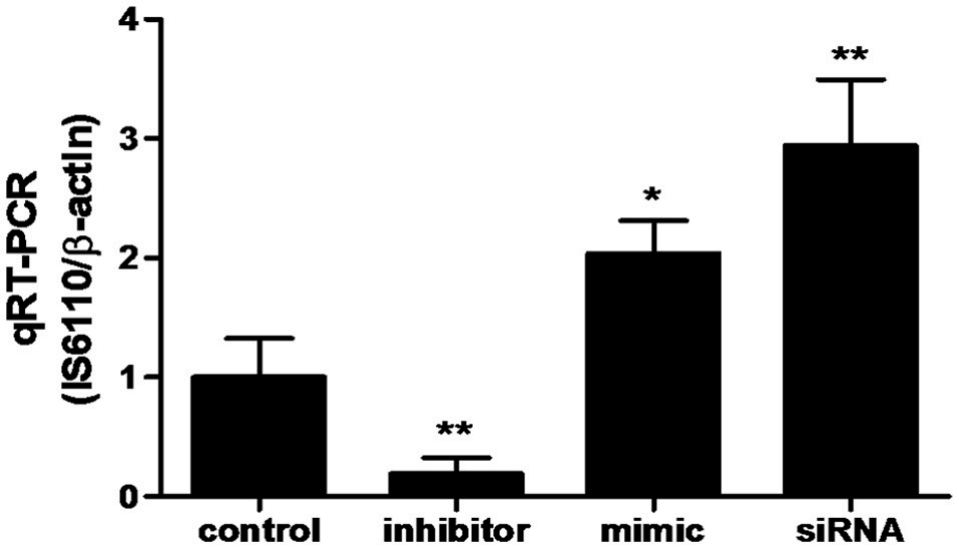
**The BCG burden in RAW264.7 cells analyzed by qRT-PCR**. The RAW264.7 cells were transfected with miR-20a control, miR-20a mimic, miR-20a inhibitor or ATG7 siRNA, and then infected with BCG at an MOI of 10 for 24 h. qRT-PCR was used to estimate the abundance of intracellular BCG in RAW264.7 cells by detecting the specific gene IS6110 of BCG. Data represent the means ± SD from three independent experiments. ^*^p < 0.05, ^**^p < 0.01.

In order to determine whether miR-20a contribute to BCG survival in macrophages by inhibiting autophagy, qRT-PCR was used to determine the bacterial load in different treatment groups. qPCR assay showed that transfection with miR-20a mimic or ATG7 siRNA significantly increased the bacterial load of intracellular BCG in RAW264.7 cells, whereas transfection with miR-20a inhibitor significantly decreased the bacterial load of intracellular BCG (Figure 6). Therefore, these results showed that miR-20a promoted BCG survival in macrophages by inhibiting autophagy.

## Discussion

Macrophages are the host cells of *M. tuberculosis* and can engulf and kill *M. tuberculosis* by initiating an inflammatory response (Liu and Modlin, 2008). Meanwhile, *M. tuberculosis* can evade immune surveillance and attacks by macrophages by various mechanisms. For example, *M. tuberculosis* prevents autophagosome fusion with lysosomes and decreases sensitivity of macrophage responses to stimulation (Liu and Modlin, 2008). Therefore, precise regulation of macrophages is crucial for maintaining mycobacterial latent infection and *M. tuberculosis* clearance. Many studies have confirmed that miRNAs contribute to *M. tuberculosis* latent infection or host evasion mechanisms (Meng et al., 2014; Kim et al., 2015; Li M. et al., 2016). Autophagy plays important roles in the host immune response against mycobacterial infection (Levine and Deretic, 2007). Recently, an increasing number of miRNAs are being discovered as key regulators in autophagy against invading pathogens including *M. tuberculosis* (Wang et al., 2013; Kumar et al., 2016; Ouimet et al., 2016). Nonetheless, the molecular mechanism of miRNA involved in autophagy-mediated mycobacterial clearance remains largely unclear. In this study, we found that miR-20a plays a novel role in inhibiting autophagy and promoting mycobacterial latent infection in macrophages by targeting ATG7 and ATG16L1, which may provide a better understanding of *M. tuberculosis* latent infection.

Following high-throughput sequencing of small RNA in RAW264.7 cells treated with rapamycin or 3-methyladenine and bioinformatics analysis, 52 miRNAs were increased more than 1.5-fold or decreased more than 2-fold. In these miRNAs, some miRNAs have been found to play specific roles in autophagic response. For example, miR-96 can promote or inhibit autophagy by principally inhibiting mTOR or ATG7 depending on the expression levels of miR-96 in prostate cancer cells under hypoxia (Ma et al., 2014). MiR-96 may also regulate autophagic pathways in macrophages, especially rapamycin-induced antophagy or 3-MA-inhibited autophagy, because the regulation of rapamycin or 3-MA on antophagy is closely related with mTOR (Wu et al., 2013). Moreover, miR-20a is a member of the miR-17-92 cluster which has been demonstrated to play important roles in various biological processes including oncogenicity (Wang Z. et al., 2015), immune regulation (Cox et al., 2010), and disease progression (Sasaki et al., 2010). It is reported that miR-20a inhibits TCR-mediated signaling and cytokine production in human naïve CD4^+^ T cells (Reddycherla et al., 2015). Zhu et al. have demonstrated that a panel of miRNAs including miR-20a, miR-17, and miR-106a can regulate macrophage inflammatory responses by targeting signal-regulatory protein α (Zhu et al., 2013). Moreover, miR-20a regulates hypoxia-induced autophagy by targeting ATG16L1 in ischemic kidney injury (Wang I. K. et al., 2015). These studies imply a potential role of miR-20a in the host immune response, which is closely related to host immune defense system against invading pathogens. An increasing number of evidence has shown that miR-20a plays an key role in autophagy, especially in cancer (Chen et al., 2016; Li S. et al., 2016). Besides, miR-20a has been shown to regulate autophagy induced by leucine deprivation in C2C12 cells via targeting ULK1 (Wu et al., 2012). These studies confirm that miR-20a can regulate autophagy by targeting various ATGs. However, the potential role of miR-20a on autophagy during *M. tuberculosis* infection remains unclear. MiR-20a is up-regulated in Rapamycin-treated RAW264.7 cells by high-throughput sequencing and in BCG-infected RAW264.7 cells by qRT-PCR analysis. These data indicate that miR-20a plays a potential role in autophagy during mycobacterial infection.

Indeed, our study showed that miR-20a could suppress the protein expression level of the ATG7 and ATG16L1. Overexpression of miR-20a inhibited ATG7 and ATG16L1 protein expression, whereas transfection with miR-20a inhibitor led to an increase in ATG7 and ATG16L1 expression in unfected or BCG-infected macrophages. Meanwhile, the target relationship of miR-20a on ATG7 and ATG16L1 mRNA 3′UTR was confirmed by luciferase reporter assays. Transfection with miR-20a mimic reduced luciferase activity in the 293T cells containing the ATG16L1-WT or ATG7-WT reporter, but failed to inhibit luciferase activity in the 293T cells containing the ATG16L1-Mut or ATG7-Mut reporter. ATG7 and ATG16L1 have been demonstrated to be essential for autophagy. ATG7 plays a pivotal role in autophagosome formation and vesicle progression. Firstly, ATG7 conjugates ATG5 to ATG12 as an E1-like ligase, which is a required step for autophagosome formation. Secondly, ATG7 converts LC3-I, an immature and cytosolic protein, into LC3-II which is a mature autophagosomal membrane protein. ATG7^−/−^ mice cannot survive within 1 day from birth owing to autophagic impairment (Komatsu et al., 2005). ATG16L1 interacts with ATG12-ATG5 to form a large protein complex essential for autophagy, which controls the elongation of the nascent autophagosomal membrane (Levine et al., 2011). Our data suggest that miR-20a regulates autophagy process by targeting ATG7 and ATG16L1 during mycobacterial infection.

Autophagy is an important element of the innate immune response against invading pathogens (Levine and Deretic, 2007). This study showed that miR-20a inhibited the accumulation of LC3 puncta in uninfected and BCG-infected RAW264.7 cells, indicating that miR-20a suppressed autophagy in macrophages during mycobacterial infection and could promote latent infection of *M. tuberculosis*. Moreover, transfection with ATG7 siRNA decreased the level of LC3-II and the number of LC3 puncta in RAW264.7 cells treated with rapamycin, and transfection with ATG7 siRNA plus miR-20a mimic could further decreased the number of LC3 puncta, indicating that miR-20a inhibits autophagy process by regulating multiple targets and multiple pathways. Quantification of autophagosomes per cellular cross-section revealed a significant reduction in RAW264.7 cells transfected with miR-20a mimic, ATG7 siRNA or miR-20a mimic plus ATG7 siRNA, whereas miR-20a downregulation increased the number of autophagosomes per cellular cross-section, confirming our results of immunofluorescence analysis. Autophagy is an effective defense mechanism preventing *M. tuberculosis* survival in macrophages through elevating the delivery of mycobacterial phagosomes (Gutierrez et al., 2004) or through enhancing the presentation of mycobacterial antigens to induce a protective CD4^+^ T lymphocyte response (Jagannath et al., 2009). Our results showed that miR-20a overexpression and ATG7 siRNA significantly increased the bacterial load of intracellular BCG in RAW264.7 cells, whereas transfection with miR-20a inhibitor significantly decreased the bacterial load of intracellular BCG.

**Figure 7:**
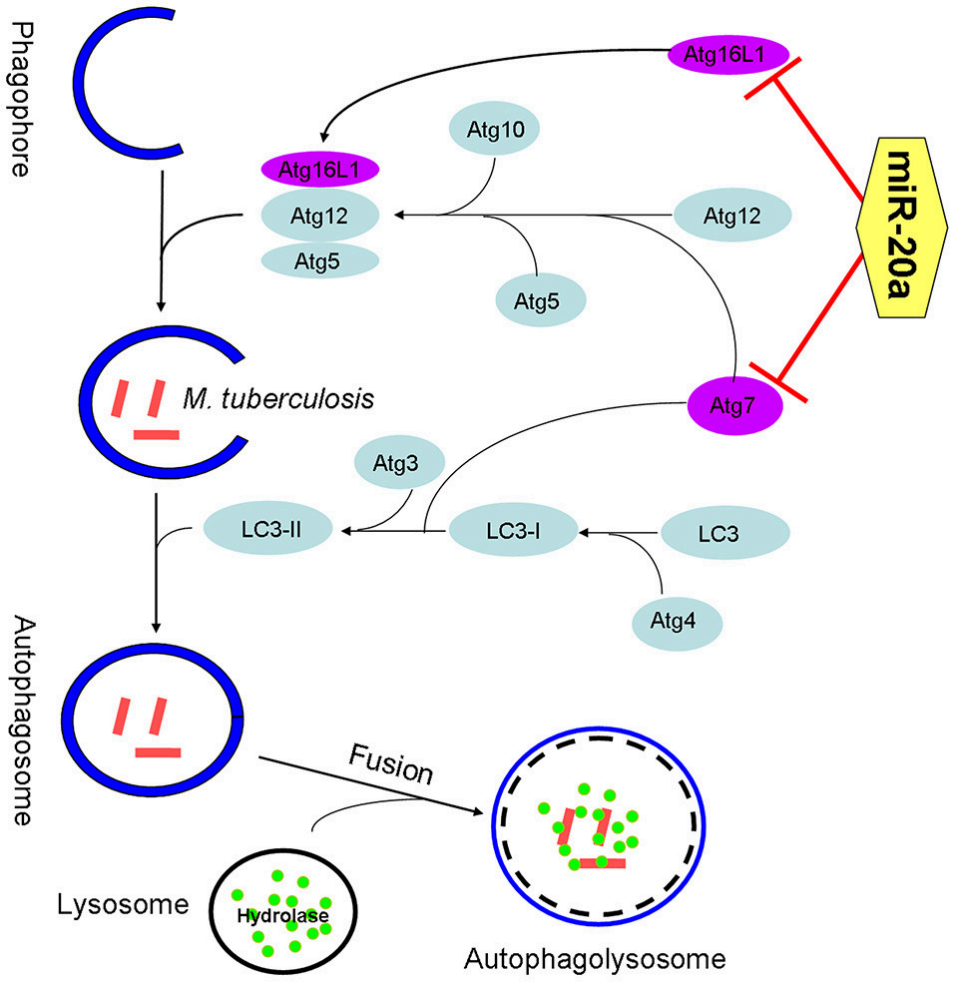
**The schematic diagram of miR-20a inhibiting antophagy process by targeting ATG7 and ATG16L1**. ATG7 is one of the master regulators of the autophagy process, responsible for two major reactions involved in autophagosome formation and in vesicle progression. Atg16L1 plays a key role in autophagosome maturation as part of a protein complex that directs LC3 to autophagosomes en route to their fusion with lysosomes. The miR-20a can inhibit antophage by targeting ATG7 and ATG16L1, and promote *M. tuberculosis* survival in macrophages.

Collectively, our data reveal that miR-20a is induced by mycobacterial infection, and suppresses the protein expression of ATG7 and ATG16L1, thereby inhibiting autophagic response and promoting latent infection of *M. tuberculosis* and survival in macrophages (Figure 7). This study reveals an important role of miR-20a in autophagy regulation, which may provide a better understanding of the mechanism by which *M. tuberculosis* could evade immune clearance and facilitate pathogenesis and persistent infection.

## Author contributions

Conceived and designed the experiments: GX and LG. Performed the experiments: JZ, YQ, QG, and SD. Analyzed the data: JZ and YQ. Contributed reagents/materials/analysis tools: JW. Wrote the manuscript: LG, YZ, and RY.

### Conflict of interest statement

The authors declare that the research was conducted in the absence of any commercial or financial relationships that could be construed as a potential conflict of interest.

## Supplementary material

Supplementary Figure 1 **High-throughput sequencing of small RNAs and Bioinformatics analysis. (A)** miRNA expression profiles. Changes of gene expression are displayed by the colored bars as higher (red) or lower (green). miR-20a was indicated by a red arrow **(B)** miRNAs with fold changes (3-MA/NC or Rapa/NC) greater than 1.5 or lower than 0.5. miR-20a was indicated by a red arrow. **(C)** The potential target genes of miR-20a were predicted by miRWalk, miRDB, miRanda and Targetscan softwares respectively. The mutual target genes were analyzed by Venny 2.1 software. **(D)** miRNAs-protein interaction network of 20 potential target genes related with autophagy and 19 selected miRNAs. Red denotes miRNAs with fold changes (3-MA/NC or Rapa/NC) greater than 1.5 or lower than 0.5. Gray nodes miRNAs with fold changes (3-MA/NC or Rapa/NC) from 0.5 to 1.5. White denotes miRNA expression with no significant change or miRNAs which are not detected by high-throughput sequencing. Solid black lines indicate the interaction relations which have been confirmed. Dotted lines denote the interaction relationships which are predicted by miRWalk software.

Supplementary Table 1 **The primers for miRNAs by qRT-PCR**.

Supplementary Data Sheet 1 **High-throughput sequencing of Small RNAs**.

## References

[B1] Chekulaeva M. Filipowicz W. (2009). Mechanisms of miRNA-mediated post-transcriptional regulation in animal cells. Curr. Opin. Cell Biol. 21, 452–460. 10.1016/j.ceb.2009.04.009 19450959

[B2] Chen J. Liu L. Liu Y. Liu X. Qu C. Meng F.. (2016). Low-dose endothelial-monocyte-activating polypeptide-II induced autophagy by down-regulating miR-20a in U-87 and U-251 glioma cells. Front. Cell. Neurosci. 10:128. 10.3389/fncel.2016.00128 27242439 PMC4868923

[B3] Cox M. B. Cairns M. J. Gandhi K. S. Carroll A. P. Moscovis S. Stewart G. J.. (2010). MicroRNAs miR-17 and miR-20a inhibit T cell activation genes and are under-expressed in MS whole blood. PLoS ONE 5:e12132. 10.1371/journal.pone.0012132 20711463 PMC2920328

[B4] Deretic V. Saitoh T. Akira S. (2013). Autophagy in infection, inflammation and immunity. Nat. Rev. Immunol. 13, 722–737. 10.1038/nri3532 24064518 PMC5340150

[B5] Fan Y. X. Dai Y. Z. Wang X. L. Ren Y. Q. Han J. J. Zhang H. (2016). MiR-18a upregulation enhances autophagy in triple negative cancer cells via inhibiting mTOR signaling pathway. Eur. Rev. Med. Pharmacol. Sci. 20, 2194–2200. Available online at: http://www.europeanreview.org/article/10908 27338042

[B6] Frankel L. B. Wen J. Lees M. Høyer-Hansen M. Farkas T. Krogh A.. (2011). microRNA-101 is a potent inhibitor of autophagy. EMBO J. 30, 4628–4641. 10.1038/emboj.2011.331 21915098 PMC3243595

[B7] Gao Y. H. Qian J. Y. Chen Z. W. Fu M. Q. Xu J. F. Xia Y.. (2016). Suppression of Bim by microRNA-19a may protect cardiomyocytes against hypoxia-induced cell death via autophagy activation. Toxicol. Lett. 257, 72–83. 10.1016/j.toxlet.2016.05.019 27220268

[B8] Gengenbacher M. Kaufmann S. H. (2012). *Mycobacterium tuberculosis*: success through dormancy. FEMS Microbiol. Rev. 36, 514–532. 10.1111/j.1574-6976.2012.00331.x 22320122 PMC3319523

[B9] Gutierrez M. G. Master S. S. Singh S. B. Taylor G. A. Colombo M. I. Deretic V. (2004). Autophagy is a defense mechanism inhibiting BCG and *Mycobacterium tuberculosis* survival in infected macrophages. Cell 119, 753–766. 10.1016/j.cell.2004.11.038 15607973

[B10] Hayashi-Nishino M. Fujita N. Noda T. Yamaguchi A. Yoshimori T. Yamamoto A. (2009). A subdomain of the endoplasmic reticulum forms a cradle for autophagosome formation. Nat. Cell Biol. 11, 1433–1437. 10.1038/ncb1991 19898463

[B11] Jagannath C. Lindsey D. R. Dhandayuthapani S. Xu Y. Hunter R. L. Jr. Eissa N. T. (2009). Autophagy enhances the efficacy of BCG vaccine by increasing peptide presentation in mouse dendritic cells. Nat. Med. 15, 267–276. 10.1038/nm.1928 19252503

[B12] Kim J. K. Yuk J. M. Kim S. Y. Kim T. S. Jin H. S. Yang C. S.. (2015). MicroRNA-125a inhibits autophagy activation and antimicrobial responses during mycobacterial infection. J. Immunol. 194, 5355–5365. 10.4049/jimmunol.1402557 25917095

[B13] Komatsu M. Waguri S. Ueno T. Iwata J. Murata S. Tanida I.. (2005). Impairment of starvation-induced and constitutive autophagy in Atg7-deficient mice. J. Cell Biol. 169, 425–434. 10.1083/jcb.200412022 15866887 PMC2171928

[B14] Korbel D. S. Schneider B. E. Schaible U. E. (2008). Innate immunity in tuberculosis: myths and truth. Microbes Infect. 10, 995–1004. 10.1016/j.micinf.2008.07.039 18762264

[B15] Kumar R. Sahu S. K. Kumar M. Jana K. Gupta P. Gupta U. D.. (2016). MicroRNA 17-5p regulates autophagy in *Mycobacterium tuberculosis*-infected macrophages by targeting Mcl-1 and STAT3. Cell. Microbiol. 18, 679–691. 10.1111/cmi.12540 26513648

[B16] Levine B. Deretic V. (2007). Unveiling the roles of autophagy in innate and adaptive immunity. Nat. Rev. Immunol. 7, 767–777. 10.1038/nri2161 17767194 PMC7097190

[B17] Levine B. Mizushima N. Virgin H. W. (2011). Autophagy in immunity and inflammation. Nature 469, 323–335. 10.1038/nature09782 21248839 PMC3131688

[B18] Li M. Wang J. Fang Y. Gong S. Li M. Wu M.. (2016). microRNA-146a promotes mycobacterial survival in macrophages through suppressing nitric oxide production. Sci. Rep. 6, 23351. 10.1038/srep23351 27025258 PMC4812255

[B19] Li S. Qiang Q. Shan H. Shi M. Gan G. Ma F.. (2016). MiR-20a and miR-20b negatively regulate autophagy by targeting RB1CC1/FIP200 in breast cancer cells. Life Sci. 147, 143–152. 10.1016/j.lfs.2016.01.044 26829385

[B20] Liu P. T. Modlin R. L. (2008). Human macrophage host defense against *Mycobacterium tuberculosis*. Curr. Opin. Immunol. 20, 371–376. 10.1016/j.coi.2008.05.014 18602003

[B21] Ma Y. Yang H. Z. Dong B. J. Zou H. B. Zhou Y. Kong X. M.. (2014). Biphasic regulation of autophagy by miR-96 in prostate cancer cells under hypoxia. Oncotarget 5, 9169–9182. 10.18632/oncotarget.2396 25333253 PMC4253426

[B22] Meng Q. L. Liu F. Yang X. Y. Liu X. M. Zhang X. Zhang C.. (2014). Identification of latent tuberculosis infection-related microRNAs in human U937 macrophages expressing *Mycobacterium tuberculosis* Hsp16.3. BMC Microbiol. 14:37. 10.1186/1471-2180-14-37 24521422 PMC3925440

[B23] Ouimet M. Koster S. Sakowski E. Ramkhelawon B. van Solingen C. Oldebeken S.. (2016). *Mycobacterium tuberculosis* induces the miR-33 locus to reprogram autophagy and host lipid metabolism. Nat. Immunol. 17, 677–686. 10.1038/ni.3434 27089382 PMC4873392

[B24] Reddycherla A. V. Meinert I. Reinhold A. Reinhold D. Schraven B. Simeoni L. (2015). miR-20a inhibits TCR-mediated signaling and cytokine production in human naive CD4+ T cells. PLoS ONE 10:e0125311. 10.1371/journal.pone.0125311 25884400 PMC4401545

[B25] Rothschild S. I. Gautschi O. Batliner J. Gugger M. Fey M. F. Tschan M. P. (2016). MicroRNA-106a targets autophagy and enhances sensitivity of lung cancer cells to Src inhibitors. Lung Cancer.. [Epub ahead of print]. 10.1016/j.lungcan.2016.06.004 27372519

[B26] Russell D. G. Barry C. E. III Flynn J. L. (2010). Tuberculosis: what we don't know can, and does, hurt us. Science 328, 852–856. 10.1126/science.1184784 20466922 PMC2872107

[B27] Sasaki K. Kohanbash G. Hoji A. Ueda R. McDonald H. A. Reinhart T. A.. (2010). miR-17-92 expression in differentiated T cells - implications for cancer immunotherapy. J. Transl. Med. 8:17. 10.1186/1479-5876-8-17 20167088 PMC2836279

[B28] Sayed D. Abdellatif M. (2011). MicroRNAs in development and disease. Physiol. Rev. 91, 827–887. 10.1152/physrev.00006.2010 21742789

[B29] Shin D. M. Jeon B. Y. Lee H. M. Jin H. S. Yuk J. M. Song C. H.. (2010). *Mycobacterium tuberculosis* eis regulates autophagy, inflammation, and cell death through redox-dependent signaling. PLoS Pathog. 6:e1001230. 10.1371/journal.ppat.1001230 21187903 PMC3002989

[B30] Sun K. T. Chen M. Y. Tu M. G. Wang I. K. Chang S. S. Li C. Y. (2015). MicroRNA-20a regulates autophagy related protein-ATG16L1 in hypoxia-induced osteoclast differentiation. Bone 73, 145–153. 10.1016/j.bone.2014.11.026 25485521

[B31] Wang I. K. Sun K. T. Tsai T. H. Chen C. W. Chang S. S. Yu T. M.. (2015). MiR-20a-5p mediates hypoxia-induced autophagy by targeting ATG16L1 in ischemic kidney injury. Life Sci. 136, 133–141. 10.1016/j.lfs.2015.07.002 26165754

[B32] Wang J. Yang K. Zhou L. Minhaowu Wu, Y. Zhu M.. (2013). MicroRNA-155 promotes autophagy to eliminate intracellular mycobacteria by targeting Rheb. PLoS Pathog. 9:e1003697. 10.1371/journal.ppat.1003697 24130493 PMC3795043

[B33] Wang Z. Wang B. Shi Y. Xu C. Xiao H. L. Ma L. N.. (2015). Oncogenic miR-20a and miR-106a enhance the invasiveness of human glioma stem cells by directly targeting TIMP-2. Oncogene 34, 1407–1419. 10.1038/onc.2014.75 24704830

[B34] Weidberg H. Shvets E. Elazar Z. (2011). Biogenesis and cargo selectivity of autophagosomes. Annu. Rev. Biochem. 80, 125–156. 10.1146/annurev-biochem-052709-094552 21548784

[B35] Wu H. Wang F. Hu S. Yin C. Li X. Zhao S.. (2012). MiR-20a and miR-106b negatively regulate autophagy induced by leucine deprivation via suppression of ULK1 expression in C2C12 myoblasts. Cell. Signal. 24, 2179–2186. 10.1016/j.cellsig.2012.07.001 22781751

[B36] Wu L. Feng Z. Cui S. Hou K. Tang L. Zhou J.. (2013). Rapamycin upregulates autophagy by inhibiting the mTOR-ULK1 pathway, resulting in reduced podocyte injury. PLoS ONE 8:e63799. 10.1371/journal.pone.0063799 23667674 PMC3648526

[B37] Yang Z. Klionsky D. J. (2010). Mammalian autophagy: core molecular machinery and signaling regulation. Curr. Opin. Cell Biol. 22, 124–131. 10.1016/j.ceb.2009.11.014 20034776 PMC2854249

[B38] Yates L. A. Norbury C. J. Gilbert R. J. (2013). The long and short of microRNA. Cell 153, 516–519. 10.1016/j.cell.2013.04.003 23622238

[B39] Zheng B. Zhu H. Gu D. Pan X. Qian L. Xue B.. (2015). MiRNA-30a-mediated autophagy inhibition sensitizes renal cell carcinoma cells to sorafenib. Biochem. Biophys. Res. Commun. 459, 234–239. 10.1016/j.bbrc.2015.02.084 25712526

[B40] Zhu D. Pan C. Li L. Bian Z. Lv Z. Shi L.. (2013). MicroRNA-17/20a/106a modulate macrophage inflammatory responses through targeting signal-regulatory protein alpha. J. Allergy Clin. Immunol. 132, 426–436.e8. 10.1016/j.jaci.2013.02.005 23562609 PMC5882493

[B41] Zullo A. J. Lee S. (2012). Mycobacterial induction of autophagy varies by species and occurs independently of mammalian target of rapamycin inhibition. J. Biol. Chem. 287, 12668–12678. 10.1074/jbc.M111.320135 22275355 PMC3339952

