# Supplementary figures and images for: microRNA-20a Inhibits Autophagic Process by Targeting ATG7 and ATG16L1 and Favors Mycobacterial Survival in Macrophage Cells
Source: Front Cell Infect Microbiol. 2016 Oct 18;6:134. doi: 10.3389/fcimb.2016.00134 (PMC5067373; doi:10.3389/fcimb.2016.00134)

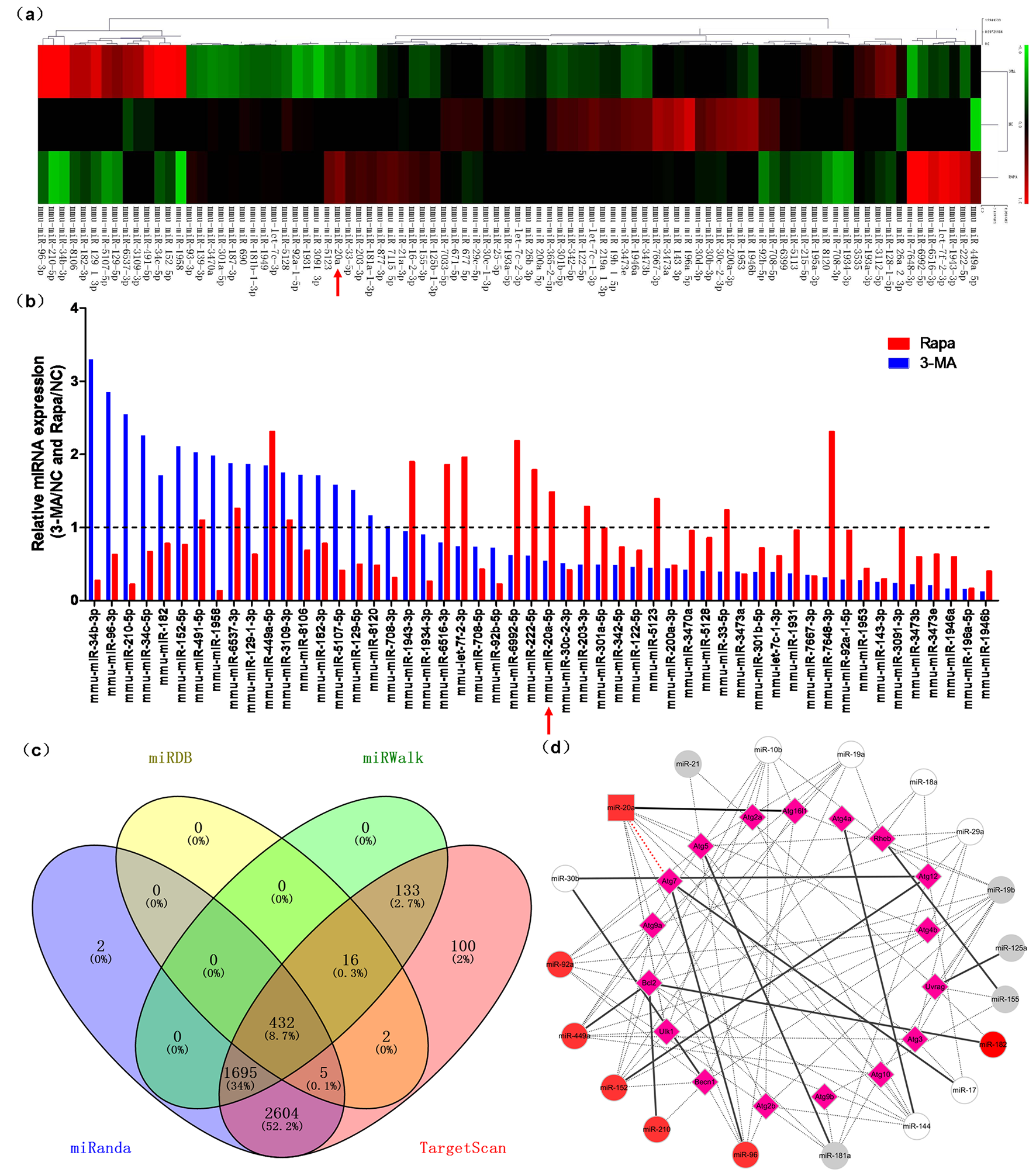

Supplement: Supplementary Figure 1 — High-throughput sequencing of small RNAs and Bioinformatics analysis. (A) miRNA expression profiles. Changes of gene expression are displayed by the colored bars as higher (red) or lower (green). miR-20a was indicated by a red arrow (B) miRNAs with fold changes (3-MA/NC or Rapa/NC) greater than 1.5 or lower than 0.5. miR-20a was indicated by a red arrow. (C) The potential target genes of miR-20a were predicted by miRWalk, miRDB, miRanda and Targetscan softwares respectively. The mutual target genes were analyzed by Venny 2.1 software. (D) miRNAs-protein interaction network of 20 potential target genes related with autophagy and 19 selected miRNAs. Red denotes miRNAs with fold changes (3-MA/NC or Rapa/NC) greater than 1.5 or lower than 0.5. Gray nodes miRNAs with fold changes (3-MA/NC or Rapa/NC) from 0.5 to 1.5. White denotes miRNA expression with no significant change or miRNAs which are not detected by high-throughput sequencing. Solid black lines indicate the interaction relations which have been confirmed. Dotted lines denote the interaction relationships which are predicted by miRWalk software. [file Image1.TIF]
