# Supplementary material for: microRNA-20a Inhibits Autophagic Process by Targeting ATG7 and ATG16L1 and Favors Mycobacterial Survival in Macrophage Cells
Source: Front Cell Infect Microbiol. 2016 Oct 18;6:134. doi: 10.3389/fcimb.2016.00134 (PMC5067373; doi:10.3389/fcimb.2016.00134)
Supplement: Supplementary Table 1 — The primers for miRNAs by qRT-PCR. [file Table1.DOC]

**Table 1. The primers for miRNAs by qRT-PCR**

| miRNAs | The sequences of primers |
| --- | --- |
| miR-17 | Stem-loop primer:  5’-CTCAACTGGTGTCGTGGAGTCGGCAATTCAGTTGAGCTACCTGC-3’  Upstream primer:  5’-ACACTCCAGCTGGGCAAAGTGCTTACAGTGCAGG-3’ |
| miR-18a | Stem-loop primer:  5’-CTCAACTGGTGTCGTGGAGTCGGCAATTCAGTTGAGCTATCTGCACTAGATG-3’  Upstream primer:  5’-ACACTCCAGCTGGGTAAGGTGCATCTAGTGCAGA-3’ |
| miR-19a | Stem-loop primer:  5’-CTCAACTGGTGTCGTGGAGTCGGCAATTCAGTTGAGTCAGTTTTGCATA-3’  Upstream primer:  ACACTCCAGCTGGGTGTGCAAATCTATGCAAAAC |
| miR-20a | Stem-loop primer:  5’-CTCAACTGGTGTCGTGGAGTCGGCAATTCAGTTGAGCTACCTGCACTATAAG-3’  Upstream primer:  5’-ACACTCCAGCTGGGTAAAGTGCTTATAGTGCAGG-3’ |
| miR-19b | Stem-loop primer:  5’-CTCAACTGGTGTCGTGGAGTCGGCAATTCAGTTGAGTCAGTTTTGCATG-3’  Upstream primer:  5’-ACACTCCAGCTGGGTGTGCAAATCCATGCAAAAC-3’ |
| miR-92a | Stem-loop primer:  5’-CTCAACTGGTGTCGTGGAGTCGGCAATTCAGTTGAGCAGGCCGG-3’  Upstream primer:  ACACTCCAGCTGGGTATTGCACTTGTCCCGGCCTG |

The downstream primers for miRNAs in Table 1 are consensus: (5’-CTCAACTGG TGTCGTGGA-3’). The primer sequences for RNU6 were: 5’-CTCGCTTCGGCAGCACA-3’ (upstream) and 5’-AACGCTTCACGAATTTGCGT-3’ (downstream).
